# Risk of mortality in patients infected with SARS-CoV-2 variant of concern 202012/1: matched cohort study

**DOI:** 10.1136/bmj.n579

**Published:** 2021-03-10

**Authors:** Robert Challen, Ellen Brooks-Pollock, Jonathan M Read, Louise Dyson, Krasimira Tsaneva-Atanasova, Leon Danon

**Affiliations:** 1 College of Engineering, Mathematics and Physical Sciences, University of Exeter, Exeter, UK; 2 Somerset NHS Foundation Trust, Taunton, UK; 3 Joint Universities Pandemic and Epidemiological Research (JUNIPER consortium); 4 University of Bristol, Bristol Veterinary School, Langford, Bristol, UK; 5 Bristol Medical School, Population Health Sciences, University of Bristol, Bristol, UK; 6 Lancaster Medical School, Lancaster University, Bailrigg, Lancaster, UK; 7 The Zeeman Institute for Systems Biology and Infectious Disease Epidemiology Research, School of Life Sciences and Mathematics Institute, University of Warwick, Coventry, UK; 8 The Alan Turing Institute, British Library, London, UK; 9 Department of Engineering Mathematics, University of Bristol, Bristol, UK

## Abstract

**Objective:**

To establish whether there is any change in mortality from infection with a new variant of SARS-CoV-2, designated a variant of concern (VOC-202012/1) in December 2020, compared with circulating SARS-CoV-2 variants.

**Design:**

Matched cohort study.

**Setting:**

Community based (pillar 2) covid-19 testing centres in the UK using the TaqPath assay (a proxy measure of VOC-202012/1 infection).

**Participants:**

54 906 matched pairs of participants who tested positive for SARS-CoV-2 in pillar 2 between 1 October 2020 and 29 January 2021, followed-up until 12 February 2021. Participants were matched on age, sex, ethnicity, index of multiple deprivation, lower tier local authority region, and sample date of positive specimens, and differed only by detectability of the spike protein gene using the TaqPath assay.

**Main outcome measure:**

Death within 28 days of the first positive SARS-CoV-2 test result.

**Results:**

The mortality hazard ratio associated with infection with VOC-202012/1 compared with infection with previously circulating variants was 1.64 (95% confidence interval 1.32 to 2.04) in patients who tested positive for covid-19 in the community. In this comparatively low risk group, this represents an increase in deaths from 2.5 to 4.1 per 1000 detected cases.

**Conclusions:**

The probability that the risk of mortality is increased by infection with VOC-202012/01 is high. If this finding is generalisable to other populations, infection with VOC-202012/1 has the potential to cause substantial additional mortality compared with previously circulating variants. Healthcare capacity planning and national and international control policies are all impacted by this finding, with increased mortality lending weight to the argument that further coordinated and stringent measures are justified to reduce deaths from SARS-CoV-2.

## Introduction

A new lineage of the SARS-CoV-2 virus (named B.1.1.7) was identified from genomic sequencing of samples from patients with covid-19 in the south east of England in early October 2020. In December 2020, Public Health England identified this virus as a variant of concern (VOC-202012/1).^1^ During December this new variant spread from the south east to London and the rest of the UK, with three quarters of infections being attributable to the new variant by 31 December 2020.^2^ The UK implemented a second national lockdown (5 November to 2 December 2020), which coincided with the relative growth of VOC-202012/1. After the lockdown, additional control measures were implemented as the increased rate of spread of the new variant became apparent and was made public.^3^ International restrictions on travel from the UK quickly followed, in particular to France and to the rest of Europe late in December 2020 to curb spread of the new variant to other countries, despite evidence that it was already present outside the UK. Since then, the prevalence of VOC-202012/1 has been observed to be increasing in both Europe and the US.^4^ ^5^ ^6^

Multiplex target polymerase chain reaction (PCR) tests used in parts of the UK national testing system can distinguish VOC-202012/1 from other SARS-CoV-2 variants. Testing using the Thermo TaqPath system in the UK has shown a close correlation between VOC-202012/1 cases confirmed by genomic sequencing and TaqPath PCR results where the spike protein gene PCR target has not been detected but other PCR targets (N gene and ORF1ab gene) have been detected.^2^ ^7^ ^9^ Such a result is referred to as S gene negative, or S gene target failure, and has a strong association to infection with the B.1.1.7 variant in the UK. S gene negative results have subsequently been used as a proxy to track the progression of this variant in the UK.^2^ ^7^ ^8^ ^9^ This association is not necessarily as strong in other countries as variants there can also produce S gene negative results.

Sequencing of VOC-202012/1 revealed 14 genetic mutations, eight of which occurred in parts of the genome that code for the spike protein responsible for cell binding,^10^ and which impairs detection of the S gene. These mutations seem to have imparted a phenotypic change to the cell binding mechanism,^2^ ^7^ ^8^ ^9^ ^11^ with the potential for increased infectivity.^12^ ^13^ The impact of the change on clinical presentation, patient outcome, and mortality remains poorly understood.

We used linked data from syndromic community testing and death records to assess whether the new SARS-CoV-2 variant is associated with a different risk of mortality compared with previously circulating variants.

## Methods

The study primarily set out to determine if mortality was different in patients testing positive for SARS-CoV-2 with PCR test results compatible with those for VOC-202012/1 compared with other variants. This objective was problematic because during the period under study rates of covid-19 cases in the UK increased steeply, putting hospital services under strain, which in turn affected mortality^14^ and potentially biased observations of mortality.

We conducted a matched cohort study. To deal with bias from the varied geographical and temporal incidence of covid-19 and its burden on hospitals we matched patients closely on time and geographical location, and we also assessed the variability of our estimates when relaxing the matching criteria.

### Inclusion criteria

People were eligible for study inclusion if they were older than 30 years and had a single positive test result for covid-19 from 1 October 2020 to 29 January 2021. We restricted our sampling to test results that reported a PCR cycle threshold value. Antigen swab tests in the UK are carried out through two routes: pillar 1 represents National Health Service testing of healthcare workers and those with a clinical need, and pillar 2 represents community testing of people with symptoms. Community based covid-19 diagnoses are generally in a younger population with less severe disease than hospital based covid-19 diagnoses, as elderly people or those with severe disease tend to present directly to hospital (see supplementary file for details). We consider only the subset of pillar 2 tests that were processed in the high throughput Lighthouse laboratories that employ the Thermo TaqPath covid-19 multiplex PCR assay, which amplifies the open reading frame 1a/b junction (ORF1ab) and the N gene and S gene of SARS-CoV-2. We included people with a single positive PCR test using the TaqPath assay and with available PCR cycle threshold values for the S, N, and ORF1ab components of SARS-CoV-2.

### Data processing

We classified SARS-CoV-2 positive test results as S gene positive (compatible with previous variants) when cycle threshold values were: S gene <30, N gene <30, and ORF1ab gene <30. We classified test results as S gene negative (compatible with VOC-202012/1) when cycle threshold values were: S gene not detected, N gene <30, and ORF1ab gene <30. Other combinations of known cycle threshold values were classified as equivocal and excluded from further analysis.

We used a unique study identifier to link the line list of positive test result details and line list of death details, when relevant. The line list of deaths records fatalities in both hospital and community settings within 28 days of a positive covid-19 test result, and follows the PHE definition of “a death in a person with a laboratory-confirmed positive covid-19 test and who died within (equal to or less than) 28 days of the first positive specimen date.”^15^ This list is maintained by PHE and represents the most timely and complete record of deaths due to covid-19 in England.^15^ The deaths line list also contains some details about the timing of hospital admission in those people who died. Patients who could not be linked and were therefore uninformative for S gene status were classified as “unknown” and were also excluded; these are generally samples not processed in Lighthouse laboratories, and include hospital cases.

During the study, hospitals experienced a period of intense demand in areas with large outbreaks of VOC-202012/01, which potentially could have adversely impacted patient outcomes. To control for any systematic bias this could have introduced, we matched people with S gene positive test results to individuals with S gene negative test results (highly likely to be VOC-202012/01) with exact matches on sex, ethnicity, index of multiple deprivation, location (as lower tier local authority region of about 190 000 people), and close matches on age (five years either way), and date of specimen collection (one day either way).

Some patients who were S gene negative matched multiple people who were S gene positive and vice versa, so we sampled participants randomly within our framework to generate 50 replicates, ensuring no S gene negative or S gene positive participant was present more than once in each replicate. All analyses were conducted on each replicate as a separate sample and the results pooled by combining the β coefficient estimates as a mixture of normal distributions and calculating combination mean and confidence intervals numerically from the mixture distribution (see supplementary file for details).

### Statistical analysis

Participants were followed-up for 28 days after infection or until 12 February 2020, after which point we censored those with no record of death. In these data more than 50% of covid-19 related deaths were reported within three days of the date of death, and more than 95% within 14 days^16^ (see supplementary file for details). The delay in reporting deaths for participants who were S gene negative and S gene positive are the same. The deaths line list is constructed from multiple sources and is considered to be the gold standard list of covid-19 related mortality in England. This list will ultimately include all deaths with covid-19 mentioned on the death certificate. We compared the rates of death in our community based dataset between participants who were S gene positive with those who were S gene negative. Using a Cox proportional hazards model we calculated the hazard ratio of death given an S gene negative test result versus death given an S gene positive test result^17^ with age (years) as a linear covariate, taking into account censoring. All analyses were performed in R (version 3.6.3).^18^ ^19^ ^20^

### Sensitivity analyses

We examined different inclusion criteria for sources of systematic bias. We systematically adjusted values for cycle thresholds for the S, N and ORF1ab genes, and the tolerances of our algorithm to match both inexact age and inexact specimen dates.

### Patient and public involvement

Owing to the nature of this research, no patients or members of the public were involved in the design or reporting of this study.

## Results

**Fig 1:**
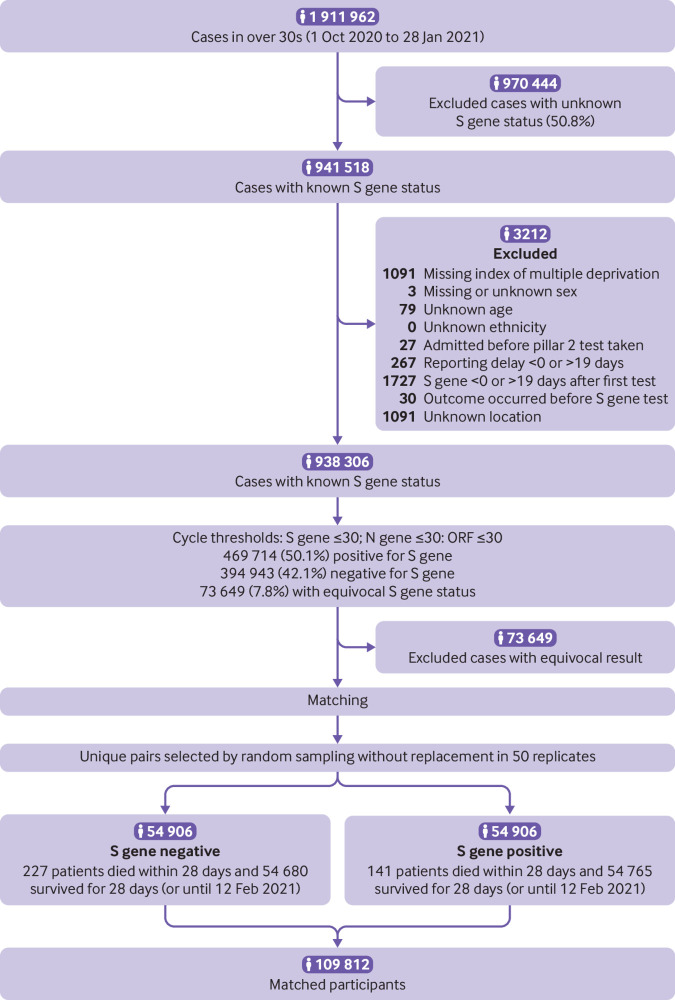
Sample selection algorithm showing average figures for numbers of participants in each study arm. Matching involved random sampling to create 50 replicates. Some cases were excluded for more than one reason

Overall, 941 518 patients older than 30 had a single positive TaqPath test result between 1 October 2020 and 28 January 2021 (fig 1). From these, 214 082 people were identified who matched with at least one other individual on age, date of specimen collection, sex, ethnicity, geographical location, and index of multiple deprivation, and differing only by S gene status. Sampling these pairs to ensure they represented unique people resulted in 50 replicates with an average of 54 906 S gene positive people and 54 906 S gene negative people in each replicate. Every person was followed-up for a minimum of 14 days after their first positive test result, and more than 85% of the cases were followed for the whole 28 day period (see supplementary file for further details). Of these 109 812 participants, 367 died (averaged over the 50 replicates) within 28 days of a positive covid-19 test result (0.3%) (table 1). The matching and sampling process is observed to control well for all personal and geographical variables considered (with slight mismatches owing to differences in scale from matching and reporting). When a tolerance of five years was allowed for matching age the average difference between study arms was 0.0 years, and when a tolerance of one day was allowed for matching specimen date a mean difference of 0.2 days was observed (with S gene negative specimens taken later than S positive specimens).

**Table 1:** Matched S gene positive and S gene negative participants. Values are numbers (percentages) unless stated otherwise

Characteristics	S gene positive (n=54 906)	S gene negative (n=54 906)	Death (n=367)
Mean (SD) age (years)	46.3 (11.0)	46.3 (11.0)	66.9 (14.1)
Age category:			
30-59	48 486 (88.3)	48 486 (88.3)	114 (31.0)
60-69	4973 (9.1)	4973 (9.1)	96 (26.1)
70-79	1175 (2.1)	1175 (2.1)	89 (24.2)
≥80	273 (0.5)	273 (0.5)	69 (18.8)
Ethnicity:			
White	45 698 (83.2)	45 698 (83.2)	325 (88.3)
Asian	6930 (12.6)	6930 (12.6)	38 (10.3)
Other	1167 (2.1)	1167 (2.1)	1 (0.3)
Unknown	127 (0.2)	127 (0.2)	
Afro-Caribbean	985 (1.8)	985 (1.8)	4 (1.1)
Sex:			
Women	29 378 (53.5)	29 378 (53.5)	141 (38.3)
Men	25528 (46.5)	25 528 (46.5)	227 (61.7)
Index of multiple deprivation 10th:			
1st	5005 (9.1)	5005 (9.1)	26 (7.0)
2nd	9413 (17.1)	9413 (17.1)	93 (25.0)
3rd	7262 (13.2)	7262 (13.2)	65 (17.5)
4th	6241 (11.4)	6241 (11.4)	34 (9.1)
5th	5344 (9.7)	5344 (9.7)	36 (9.7)
6th	4402 (8.0)	4402 (8.0)	31 (8.3)
7th	4421 (8.1)	4421 (8.1)	24 (6.5)
8th	4336 (7.9)	4336 (7.9)	26 (7.0)
9th	4364 (7.9)	4364 (7.9)	20 (5.4)
10th	4123 (7.5)	4123 (7.5)	17 (4.6)
Mean (SD) N gene cycle threshold	21.3 (4.2)	19.0 (4.4)	18.3 (4.3)
Region:			
East of England	3634 (6.6)	3637 (6.6)	18 (4.9)
London	8874 (16.2)	8874 (16.2)	26 (7.0)
Midlands	10 550 (19.2)	10 563 (19.2)	88 (23.7)
North East and Yorkshire	10 733 (19.5)	10 740 (19.6)	83 (22.4)
North West	14 711 (26.8)	14 693 (26.8)	123 (33.2)
South East	5105 (9.3)	5106 (9.3)	22 (5.9)
South West	1301 (2.4)	1297 (2.4)	11 (3.0)
S gene:			
Positive	54 906 (100.0)		141 (38.3)
Negative		54 906 (100.0)	227 (61.7)
Status:			
Dead <28 days of positive covid-19 result	141 (0.3)	227 (0.4)	367 (100.0)
Survived 28 days or until 12 Feb 2020	54 765 (99.7)	54 680 (99.6)	

Participants were matched on age, ethnicity, sex, index of multiple deprivation, geography, and specimen date (not shown).

The subset of participants who died were generally older (mean 66.9 *v* 46.3 years) and a higher proportion were men, as has been reported previously.^21^ Both cases and deaths were underrepresented in the south west and east of England—these areas had only recently used TaqPath assays and thus did not report S gene status.

**Fig 2:**
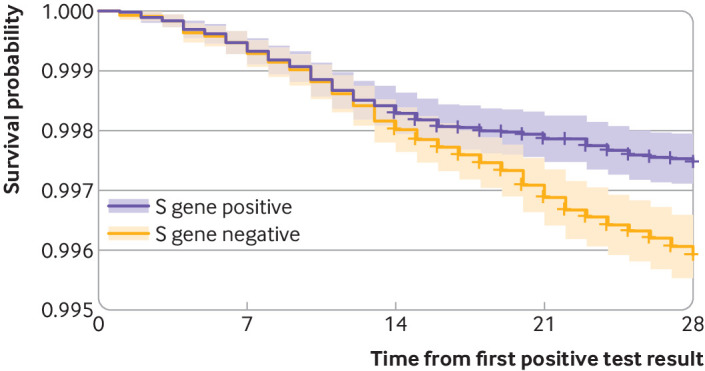
Kaplan-Meier survival curve for S gene positive (previously circulating variants) and S gene negative (new variant VOC-202012/1) participants in the UK. The y axis has been truncated as mortality was low in both groups

Of the 54 906 participants in the S gene negative arm, an average of 227 deaths occurred compared with 141 of 54 906 in the S gene positive arm (hazard ratio 1.64, 95% confidence interval 1.32 to 2.04; P<0.001) over the study period (table 2). The rate of death of S gene negative and S gene positive participants diverged after 14 days (fig 2). The proportional hazards assumption of the Cox model was therefore violated as the hazard ratio was not constant over time. This was investigated further (see supplementary file), and the violation might be corrected by considering the hazard ratio in days 0 to 14 compared with days 15 to 28 of follow-up. The hazard ratio in the first period was not significantly increased, but in days 15 to 28 the hazard ratio was 2.40 (1.66 to 3.47).

**Table 2:** Risk of death in S gene negative compared with S gene positive (reference category) participants

Model, predictor, value	Hazard ratio (95% CI)	P value
**S gene+age**		
S gene status:		
Positive (ref)	—	—
Negative	1.64 (1.32 to 2.04)	<0.001
Age (per decade)	3.55 (3.28 to 3.84)	<0.001
**S gene+N gene cycle threshold+age**		
S gene status:		
Positive (ref)	—	—
Negative	1.37 (1.09 to 1.72)	0.004
Age (per decade)	3.51 (3.24 to 3.80)	<0.001
N gene cycle threshold (per 10 units)	0.50 (0.39 to 0.65)	<0.001

Hazard ratios >1 are indicative of an increased rate of death in people with infections compatible with VOC-202012/01. In the first model the S gene status is assessed as an indicator with age as a covariate, in the second model variability is included in the N gene cycle threshold value measured in the original specimen as a continuous predictor.

**Fig 3:**
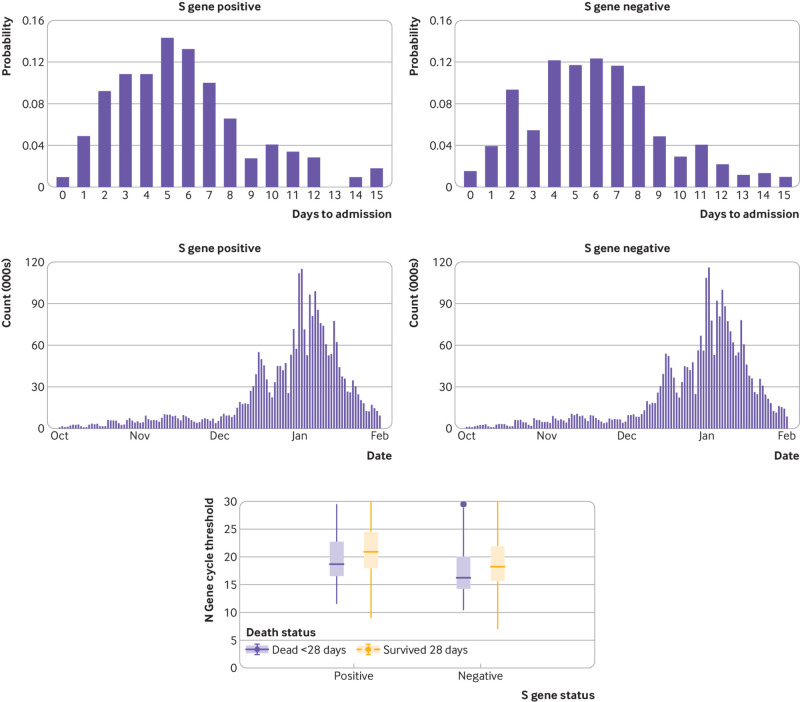
Investigation of biases in S gene positive and S gene negative study arms

The matched cohort design controls for most potential biases, including variations in hospital capacity, as it pairs patients by personal characteristics, geography, and time of testing. Other further potential biases that might be present were investigated. One possibility for bias could be a difference in the timing of presentation of S gene negative and S gene positive people for testing, with, for example, S gene positive people presenting earlier, and thus seeming to progress slower. Hospital admission data were only available for patients who ultimately died, but there was no evidence for asymmetrical delays in time from test to hospital admission (fig 3). The Office for National Statistics also investigated this and found that S gene negative patients are more likely to present earlier for testing.^22^

The paired cases in this study were spread over time but concentrated around the end of December 2020 and beginning of January 2021 (fig 3). As the ratio of S gene negativity to S gene positivity changed over this period, in the early stages it was comparatively difficult to match S gene negative people with S gene positive equivalents, and in the later stages it was difficult to match S gene positive people with S gene negative equivalents, with the bulk of matching occurring during the time of transition from dominance of the S gene positive variant to dominance of the S gene negative variant (see supplementary file).

Cycle threshold values for the N gene were lower in participants who were S gene negative than in those who were S gene positive, and this effect was potentiated in those who died (table 1 and fig 3). Low values for the N gene cycle threshold implied that the viral load in participants at the time of sampling was higher. The higher mortality could be associated with the higher viral load in S gene negative participants because of the intrinsic properties of the VOC202012/1 mutation. Alternatively, it could be an indication of the timing of testing, with people who were S gene negative presenting at peak infectiousness, for some as yet unknown reason. Thus, cycle threshold values for the N gene could be regarded either as an indication of bias or as a feature of S gene negative infection. If this is interpreted as a source of bias, the Cox proportional hazards model can control for the N gene cycled threshold value (table 2, second model), which for S gene negativity showed a hazard ratio of 1.37 (95% confidence interval 1.09 to 1.72). Even if increased viral load as a biological feature of S gene negative infection is not considered, the residual increase in hazard ratio implies a mortality effect not explained by viral load alone.

### Sensitivity analysis

**Fig 4:**
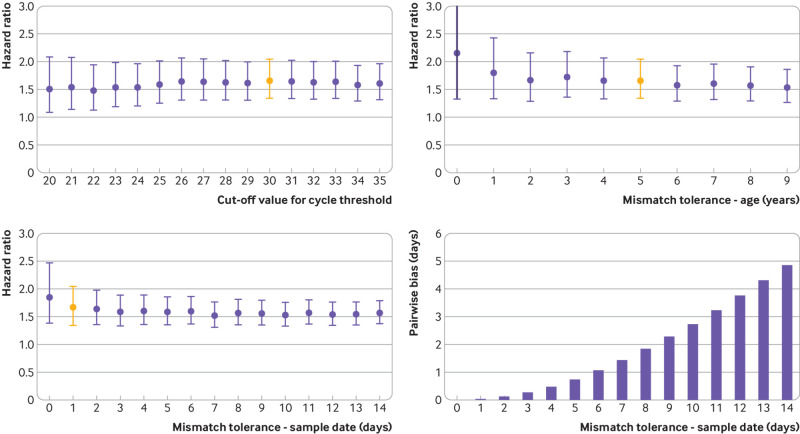
Sensitivity analyses. Red bar indicates default assumptions (cycle threshold <30; age tolerance ±5 years; sample date tolerance ±1 day) from rest of study

The cut-off value of cycle threshold used in definition of gene positivity mildly affects the central estimate of the hazard ratio, such that when the cycle threshold value for identifying a particular gene was reduced, the central estimate of hazard ratios was observed to decrease (fig 4). Lower cycle threshold values were associated with a reduction in the number of certain S gene positive and S gene negative results and an increase in the number of equivocal results, which were subsequently excluded from analysis, resulting in an effective reduction in overall case numbers. Given that cycle threshold values were generally higher in patients with S gene positivity, as defined at the cycle threshold value of less than 30, further reductions in the cut-off value of the cycle threshold tend to be associated with a reclassification of S gene positive rather than S gene negative patients with more mild disease as equivocal. This could explain the small reduction in hazard ratio associated with reducing the cut-off value of the cycle threshold. A marginal, non-significant increase was observed at a cycle threshold cut-off value of 30; as most laboratories use this as a standard cycle threshold cut-off value, this was chosen as the central estimate.

When matching patients for the cohort, allowing larger mismatching led to small changes in the associated hazard ratio estimate. The effect of mismatching on age between S gene negative and S gene positive participants did not create a systematic bias, and the mean age difference between both study arms was less than 0.005 years (fig 4). Age was observed to be a strong predictor of mortality in covid-19, so some potential bias might be expected; this is controlled for by including age as a covariate in the calculation of hazard ratios (table 2).

A dilution of hazard ratios was observed when a greater degree of mismatching was allowed between sample dates in S gene positive patients matched to S gene negative patients (fig 4). Because of the change in prevalence over the study period from predominantly S gene positive to predominantly S gene negative, increasing the degree of mismatching of sample date was associated with a systematic pairwise bias in the dates of the original positive test result (fig 4), with S gene negative patients generally being identified after S gene positive patients. Given that over the study period the number of cases was observed to exponentially increase, this could have affected the overall result as hospital capacity generally worsened during the study period. To avoid this the study minimised the sample date tolerance, trading off the reduction in bias against the variance introduced by the reduced number of cases resulting from tight matching criteria.

Despite the differences between the combinations investigated, all studies reported a statistically significant increase in the risk of mortality associated with VOC-202012/1, suggesting a real effect, and most central estimates were within the range of 1.5 to 1.7. The supplementary file discusses other potential covariates.

## Discussion

Infections with the new variant VOC-202012/1 (as measured by S gene negativity) were associated with an increased risk of death (P<0.001) in people testing positive for covid-19 in the community. The increased hazard ratio between 1.32 and 2.04, higher than for other variants, translates to a 32% to 104% increased risk of death, with the most probable hazard ratio estimate of 1.64, or a 64% increased risk of death. The absolute risk of death in this group of community identified participants, however, remains relatively low, increasing from 2.5 to 4.1 deaths per 1000 cases.

We controlled for several biases when using a matched cohort approach. In particular, mortality is affected by how many patients require intensive care in a hospital setting^14^; increasing numbers of patients in the study period (1 October 2020 to 12 February 2021), compounded by staff absenteeism as a result of covid-19 infection or isolation because of contact with infected people, has placed intense strain on hospital services and a reduction in the staff to patient ratio. Staff absenteeism might have affected mortality and is a potential source of bias. We controlled for this by matching patients on administrative region and time of positive test result (within one day), which constrains pairs to receive care at the same place and time, and we suggest at a similar level of care. Although age related mortality is controlled for by matching on age (within five years), it is also controlled for by using the Cox proportional hazards model.

As this was a community based study, we do not have information on the S gene status of patients in hospitals. The community based testing (pillar 2) in this dataset covered a younger age group and hence represented less severe disease than patients detected through hospital based testing (pillar 1). Death remains a comparatively rare outcome in patients detected in the community compared with identified in-hospital deaths. Our study only includes about 8% of the total deaths that occurred during the study period. Of all coronavirus deaths, about 26% occurred in those who were identified in the community, and data on S gene status was only available for 30%.^23^ Whether the increase in mortality from community based testing is also observed in elderly patients or in patients admitted to hospital remains to be seen.

We cannot exclude a selection bias. Community testing is largely self-selected, or driven by contact tracing. A potential bias remains if a higher proportion of patients with S gene negative infections without symptoms were undetected than patients with S gene positive infections. In this event, patients infected with VOC-202012/1 might be at a more advanced stage of disease when identified and have a higher apparent mortality. This could be consistent with the lower N gene cycle threshold values observed in S gene negative participants. Our analysis, or any retrospective study based on patients with symptoms, would not be able to detect this; however, early survey data suggest that people with S gene negative infections are, if anything, more likely to present for testing.^22^ Dealing with this potential bias requires a study design capable of detecting asymptomatic infections in participants who are negative or positive for the S gene.

Some of the increased risk could be explained by comorbidities. Information was not available about comorbid conditions in the data we analysed, although this would be partly controlled for by matching on age, ethnicity, and index of multiple deprivation. Currently there is no evidence of a mechanistic reason why people with certain comorbidities would be infected with one variant and not another. It is possible, however, that people with certain comorbidities are at a higher risk of infection with VOC-202012/1 and have a higher mortality rate. This would tend to reduce the hazard ratio attributable to VOC-202012/1 alone.

Our preliminary estimate of the hazard ratio was 1.91 (95% confidence interval 1.35 to 2.71), which is marginally higher than the estimate presented here with compatible uncertainty.^23^ ^24^ This was based on 94 deaths in S gene negative patients and 49 deaths in S gene positive patients in 66 208 less strictly matched pairs, with a shorter study period, and limited follow-up. As the new variant outbreak has unfolded and more data have become available, we have been able to obtain more accurate central estimates by narrowing the tolerance for mismatches, extending the study period and increasing the proportion of patients with complete follow-up. The design of this study is well suited to determining, in an unbiased manner, whether the risk of death has increased, although we studied a comparatively small number of patients. Other study designs, involving the use of unpaired samples, might be better able to quantify the absolute increase in risk, albeit with more potential for bias.^25^ Other recent studies produced similar estimates of the increased hazard ratio. Although these studies use the same community based testing data, they had different study and analysis designs. The preliminary results of these studies were compatible point estimates of the mortality hazard ratio (1.3 to 1.65), and the confidence intervals of these studies overlap with those described here.^23^ As with our work, these other estimates are being continuously re-evaluated as more data are acquired; and in subsequent updates some of these have been revised upwards.^26^

### Conclusions

The variant of concern, in addition to being more transmissible, seems to be more lethal. We expect this to be associated with changes in its phenotypic properties because of multiple genetic mutations,^27^ and we see no reason why this finding would be specific to the UK. This development, borne out in epidemiological analyses, implies that the rate of patients with serious infection requiring hospital attention will increase. At the time of writing (15 February 2021) the national lockdown appears to be effective at reducing the transmission rate of SARS-CoV-2 in the UK, but proliferation of the new variant has made it more difficult to control the covid-19 outbreak. The resulting number of deaths will scale linearly with the proportion of people infected with the new variant. Other analyses have indicated that the new variant is also associated with increased transmissibility, which would lead to a potentially exponential increase in the resulting number of deaths.^12^ Clinicians at the front line should be aware that a higher mortality rate is likely even if quality of practice remains unchanged. This has broader implications for any vaccination allocation policy designed to reduce mortality in the late middle age groups, typical of the community identified patients in this dataset.

The question remains whether excess mortality due to VOC-202012/1 will be observed in other population groups, particularly elderly people, care home residents, and those with other comorbidities who generally present directly to hospital as an emergency. Hospital based studies require a mechanism to distinguish emerging variants from previously circulating variants, currently only done through genotyping. Owing to the effort involved, the proportion of genotyped samples representing patients admitted to hospital remains low, and we recommend that PCR tests that specifically target VOC-202012/1 mutations should be more widely used.

Moreover, the emergence of VOC-202012/1 and its mutations (including E484K), combined with other variants of concern, including those identified in Brazil and South Africa,^28^ highlights the capacity of SARS-CoV-2 to rapidly evolve new phenotypic variants, with mutants that evade vaccines being a real possibility.^29^ Our study has helped to characterise the clinical presentation and outcome of one new variant, but given sufficient amounts of informative data our findings can be generalisable to other variants. Assessment of the clinical outcomes of multiple circulating phenotypic variants, however, requires scalable technology that is capable of identifying substantial numbers of patients infected with emerging variants (eg, broad PCR assay panels targeting variant foci^30^) and robust collection of outcome data.

In this study we controlled for the effect of time, geographical location, age, sex, ethnicity, and deprivation, but these are important factors to understand if future outcomes are to improve. Future work on the relative impact of these might allow for better targeting of resource allocation,^31^ vaccine distribution strategies, and relaxation of restrictions.

**What is already known on this topic**

The SARS-CoV-2 variant of concern 202012/1, first detected in the south east of England in autumn 2020, is more transmissible than previously circulating variants
The emergence of this variant coincided with high hospital occupancy, which is known to increase mortality
Before this study, unbiased estimates of the mortality of the variant of concern were not available

**What this study adds**

Individuals infected with the variant of concern, identified at UK community test centres, were between 32% and 104% (central estimate 64%) more likely to die than equivalent individuals infected with previously circulating variants
The absolute risk of death in this largely unvaccinated population remains low, but clinicians and public health officials should be aware that a higher mortality rate is likely even if practice remains unchanged

## Web extra

Extra material supplied by authors

Supplementary information: Additional material supplied by authors

## Notes

Contributors: RC and LDanon designed the study. All authors conceived the article. RC analysed the data and wrote the initial draft of the manuscript. KTA, EB-P, LDanon, JMR, and LDyson commented on, and revised, the manuscript. All authors read and approved the final manuscript. RC is the guarantor. The views presented here are those of the authors and should not be attributed to Somerset Foundation NHS Trust or Global Digital Exemplar. The corresponding author attests that all listed authors meet authorship criteria and that no others meeting the criteria have been omitted.

Ethical approval: The data were supplied from the Second Generation Surveillance System (SGSS) database and death reports after anonymisation under strict data protection protocols agreed between the University of Exeter and Public Health England. The ethics of the use of these data for these purposes was agreed by Public Health England with the UK government SPI-M(O)/SAGE committees. The research was assessed as not needing NHS research ethics committee review.

Data sharing: The data analysis code is available at doi:10.5281/zenodo.4543510. Data are available to members of universities who have data sharing agreements in place with Public Health England.

The lead author (RC) affirms that the manuscript is an honest, accurate, and transparent account of the study being reported; that no important aspects of the study have been omitted; and that any discrepancies from the study as planned (and, if relevant, registered) have been explained.

Dissemination to participants and related patient and public communities: Direct dissemination to study participants is not possible. Preprint versions of this analysis are available on *MedRxiv* (www.medrxiv.org/content/10.1101/2021.02.09.21250937v2).

Provenance and peer review: Not commissioned; externally peer reviewed.
